# Curcumin promotes oligodendrocyte differentiation and their protection against TNF-α through the activation of the nuclear receptor PPAR-γ

**DOI:** 10.1038/s41598-021-83938-y

**Published:** 2021-03-02

**Authors:** Antonietta Bernardo, Cristina Plumitallo, Chiara De Nuccio, Sergio Visentin, Luisa Minghetti

**Affiliations:** 1 Department of Cell Biology and Neurosciences, Istituto Superiore di Sanità, Viale Regina Elena 299, 00161 Rome, Italy; 2 Present Address: National Center for Research and Preclinical and Clinical Evaluation of Drugs, Istituto Superiore di Sanità, Viale Regina Elena 299, 00161 Rome, Italy; 3 Present Address: Research Coordination and Support Service, Istituto Superiore di Sanità, Viale Regina Elena 299, 00161 Rome, Italy

**Keywords:** Oligodendrocyte, Glial biology, Cellular neuroscience

## Abstract

Curcumin is a compound found in the rhizome of Curcuma longa (turmeric) with a large repertoire of pharmacological properties, including anti-inflammatory and neuroprotective activities. The current study aims to assess the effects of this natural compound on oligodendrocyte progenitor (OP) differentiation, particularly in inflammatory conditions. We found that curcumin can promote the differentiation of OPs and to counteract the maturation arrest of OPs induced by TNF-α by a mechanism involving PPAR-γ (peroxisome proliferator activated receptor), a ligand-activated transcription factor with neuroprotective and anti-inflammatory capabilities. Furthermore, curcumin induces the phosphorylation of the protein kinase ERK1/2 known to regulate the transition from OPs to immature oligodendrocytes (OLs), by a mechanism only partially dependent on PPAR-γ. Curcumin is also able to raise the levels of the co-factor PGC1-α and of the cytochrome c oxidase core protein COX1, even when OPs are exposed to TNF-α, through a PPAR-γ-mediated mechanism, in line with the known ability of PPAR-γ to promote mitochondrial integrity and functions, which are crucial for OL differentiation to occur. Altogether, this study provides evidence for a further mechanism of action of curcumin besides its well-known anti-inflammatory properties and supports the suggested therapeutic potential of this nutraceutical in demyelinating diseases.

## Introduction

Curcumin [1.7-bis (4-Hydroxy-3-methoxyphenyl)-1.6-heptadiene-3.5-dione], is the main polyphenol found in the rhizome of Curcuma longa (turmeric) and other Curcuma plants and is commonly used as a dietary component and supplement worldwide^1^.

A large number of studies in the last decades have suggested beneficial actions of curcumin in several pathologies and their experimental models, including neurodegenerative diseases, such as Alzheimer's disease^2–4^ and neuroinflammatory diseases, such as multiple sclerosis^5–7^.

The multiple activities of curcumin are likely due to its ability to interact indirectly with numerous transcription factors, including nuclear factor-kappa B (NF-κB), activator protein 1 (AP-1), β-catenin, signal transducer and activator of transcription (STAT) proteins, and to act as a partial agonist of the peroxisome proliferator-activated receptor-γ (PPAR-γ), a ligand-activated transcription factor involved in both neuroprotective and anti-inflammatory signaling pathways^8–12^.

PPAR-γ is part of the large superfamily of nuclear receptors comprising receptors for steroids, thyroid hormones, and retinoids, involved in the control of reproduction, metabolism, development, and immune response.

In the last years, we have focused our attention on the identification of new compounds able to activate PPAR-γ but devoid of the unwanted side effects that have been described for some classes of synthetic PPAR-γ agonists^13^. Our previous studies have shown that similarly to synthetic PPAR-γ agonists^14–16^, natural partial agonists such as the essential omega-3 fatty acid Docosahexaenoic acid (DHA) and the nonenzymatic oxygenated metabolites of α-linolenic acid B1- and L1-phytoprostanes can promote the differentiation of oligodendrocyte (OLs), favoring their maturation and protecting them from inflammatory and oxidative damage^17–19^.

The importance of identifying strategies capable of restoring OL functionality has gathered significant interest, considering the vulnerability of this cell type, responsible for the generation and maintenance of myelin in the central nervous system, to inflammatory and oxidative damage^20^.

Myelin degeneration and failure of axonal remyelination are central to major human diseases, including multiple sclerosis. These pathologic processes are associated with OL damage, leading to their death or to their inability to accomplish their full differentiation, forming the myelin sheet and restoring neuronal functions. OLs and their immature progenitors are, therefore, significant targets for therapeutic strategies for treating demyelinating diseases.

With this background, in the present study, we used primary oligodendrocyte progenitor (OP) cultures as an experimental model to test whether the suggested beneficial effect of curcumin in myelin diseases could be exerted also through its partial PPAR-γ agonistic activity. Besides, we investigated whether curcumin could thus protect OLs in experimental conditions that can mimic demyelinating diseases.

## Results

### Effect of curcumin on OP viability and differentiation: role PPAR-γ activation

**Figure 1:**
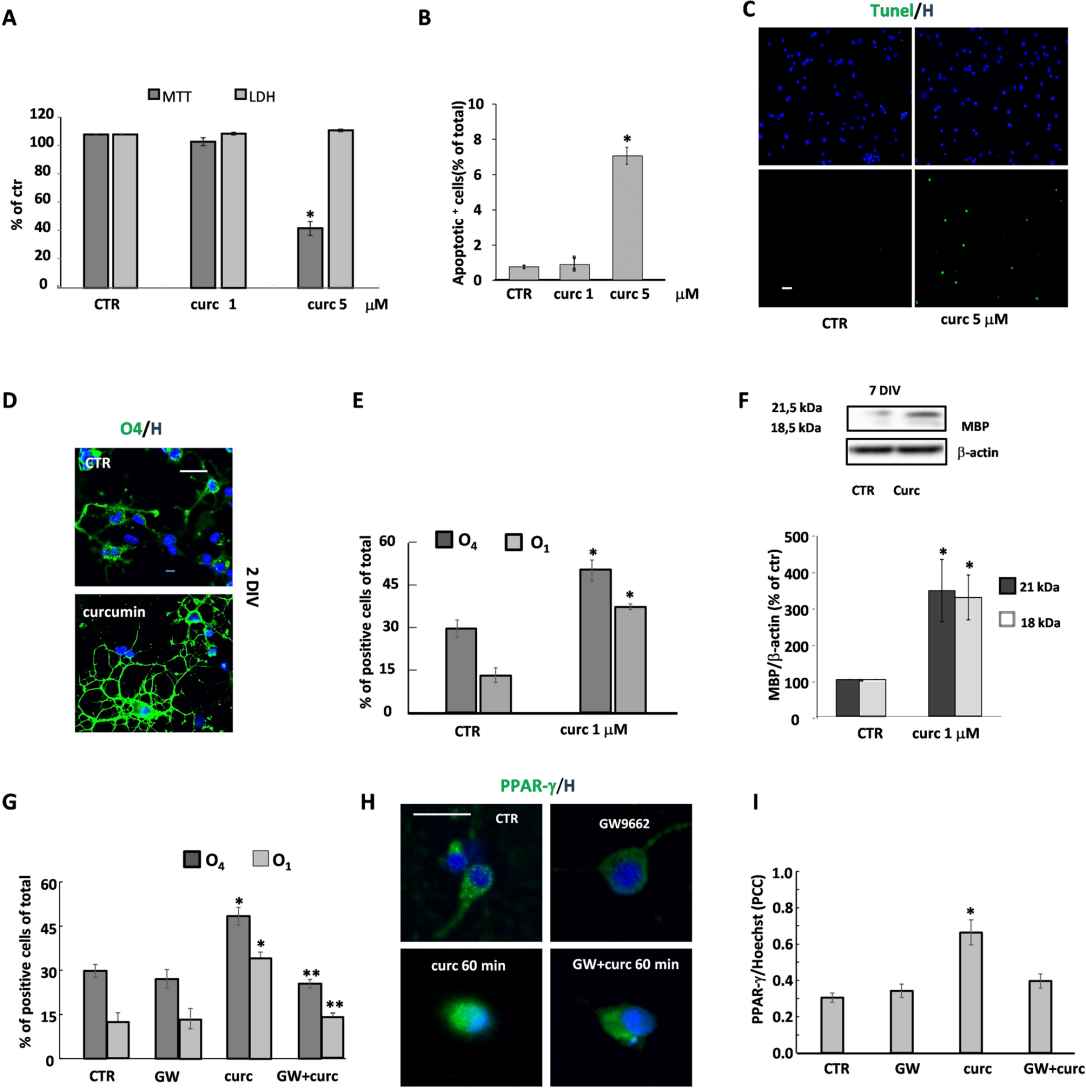
Effects of curcumin on OP viability and differentiation. The effects of 24 h treatment with curcumin (curc, 1 or 5 μM) on OP viability were examined using the MTT assay and the LDH level production. The values (means ± SEM) from 3 to 5 independent experiments run in triplicate are shown in (**A**). The number of apoptotic cells was evaluated in OP cultures exposed to 1 or 5 μM curcumin. Total cell numbers were assessed by staining with Hoechst 33258 vital dye and apoptotic cells were identified by nuclear morphology (**B**) or by Tunel assay (green labelling, **C**). Data of both tests are included, and the percentages of total cell number in each condition are showed. Labelled cells were counted in 6 to 10 coverslips from 3 to 5 independent experiments (**B**). *p < 0.0001 vs CTR. After 24 h of curcumin treatment (1 μM), OPs were immunostained for the two developmental markers O_4_ and O_1_. Nuclei were marked using the Hoechst 33258 (H). Panel (**D**) shows O_4_ (green) and H (blue) labelling. Cells were counted and percentages of O_4_- and O_1_-positive cells are shown in (**E**). Data are mean ± SEM of 10 microscopic fields per coverslip prepared in duplicate from 3 to 5 independent experiments. At 7 DIV (**F**) the expression of MBP was evaluated by western blot analysis. Representative western blot of two isoforms (18 e 21 kDa) of MBP and β-actin are shown (top panel in **F**). Bar graphs show the quantitative evaluation of MBP/β-actin expression ratio of both isoforms from three different experiments (bottom panel in **F**). To verify the involvement of PPAR-γ, OPs at 1 DIV were treated for 24 h with curc (1 μM) in the presence or absence of the PPAR-γ antagonist GW9662 (GW) (1 μM) and the percentages of O_4_ and O_1_ positive cells were evaluated (**G**). Data are mean ± SEM of 3 to 5 independent experiments *p < 0.001 versus CTR, **p < 0.001 versus curc. The translocation of PPAR-γ in the presence/absence of curcumin and GW9662 was evaluated by immunofluorescence. Panel (**H**) shows PPAR-γ (green) and H (blue) labelling. The colocalization of PPAR-γ and Hoechst fluorescence signal was evaluated by Pearson’s correlation coefficients (PCC) where PCC = 1 corresponds to maximal colocalization. Data are mean ± SEM of 3 independent experiments. Scale bar = 30 µm. Image Lab 4.0 software (Bio-Rad, http://www.bio-rad.com/en-us/sku/1709690-image-lab-software) and Leica Application Suite Software (260RI, https: //www.leica-microsystems.com/products/microscope-software/details/product/leica-las-x-ls/) were used respectively for Wb and IF image acquisitions.

All experiments were performed in highly purified cultures of rat OPs. At 1 day in vitro (DIV), cells were treated for 24 h, with curcumin at two different concentrations and cell viability evaluated by testing cell metabolic activity (MTT test), damage (LDH test) and apoptosis (Tunel assay). Curcumin markedly decreased OP metabolic activity at the highest concentration (5 μM), while none of the two concentrations affected LDH levels in the supernatants (Fig. 1A). Only at the highest concentration, curcumin induced a slight increase in the number of apoptotic cells (Fig. 1B, C). Tunel quantification data were comparable to those from morphological analysis (Fig. 1C): CTR 0.82 ± 0.08, curc 1 μM 0.93 ± 0.09, curc 5 μM 7.1 ± 0.4, assessed as % of total.

Based on these preliminary experiments, OPs were treated with 1 μM curcumin at 1 DIV. The effects on differentiation were investigated at 2 DIV and 7 DIV, by evaluating markers of specific stages of differentiation, such as O_4_ (pre-OL), O_1_ (immature OL) and myelin basic protein (MBP) (non-myelinating mature OL). As indicated in Fig. 1D, curcumin induced a drastic change in cell morphology, as depicted by the more complex ramifications of branches as compared to untreated cultures. Along with the morphological variation, curcumin treatment induced an increase in the number of O_4_ and O_1_ positive cells comparing to untreated cultures (Fig. 1E). Moreover, at 7DIV, the expression of the two isoforms of myelin basic protein (MBP) was increased, further indicating the differentiation-promoting effect of curcumin (Fig. 1F). To verify whether curcumin acted through the activation of the nuclear receptor PPAR-γ, cell differentiation was evaluated in the presence of the PPAR-γ antagonist GW9662 (1 μM). As shown in Fig. 1G, the increased number of O_4_ and O_1_-positive cells induced by treatment with curcumin was reduced at control levels by GW9662. Furthermore, curcumin acted as bona fide PPAR-γ agonist, being able to promote the nuclear translocation of PPAR-γ; curcumin-induced PPAR-γ translocation was abolished by the PPAR-γ antagonist GW9662 (Fig. 1H, I).

### Curcumin modulates ERK1/2 signaling pathways

Recent studies have shown that, in addition to the induction or repression of target genes, PPAR-γ can perform "non-genomic" actions, including activation or inhibition of several kinases^21^. Among such kinases is the protein kinase ERK (Extracellular Receptor-activated Kinase) 1/2, which is involved in the differentiation of OLs, especially in the early stages^22^.

**Figure 2:**
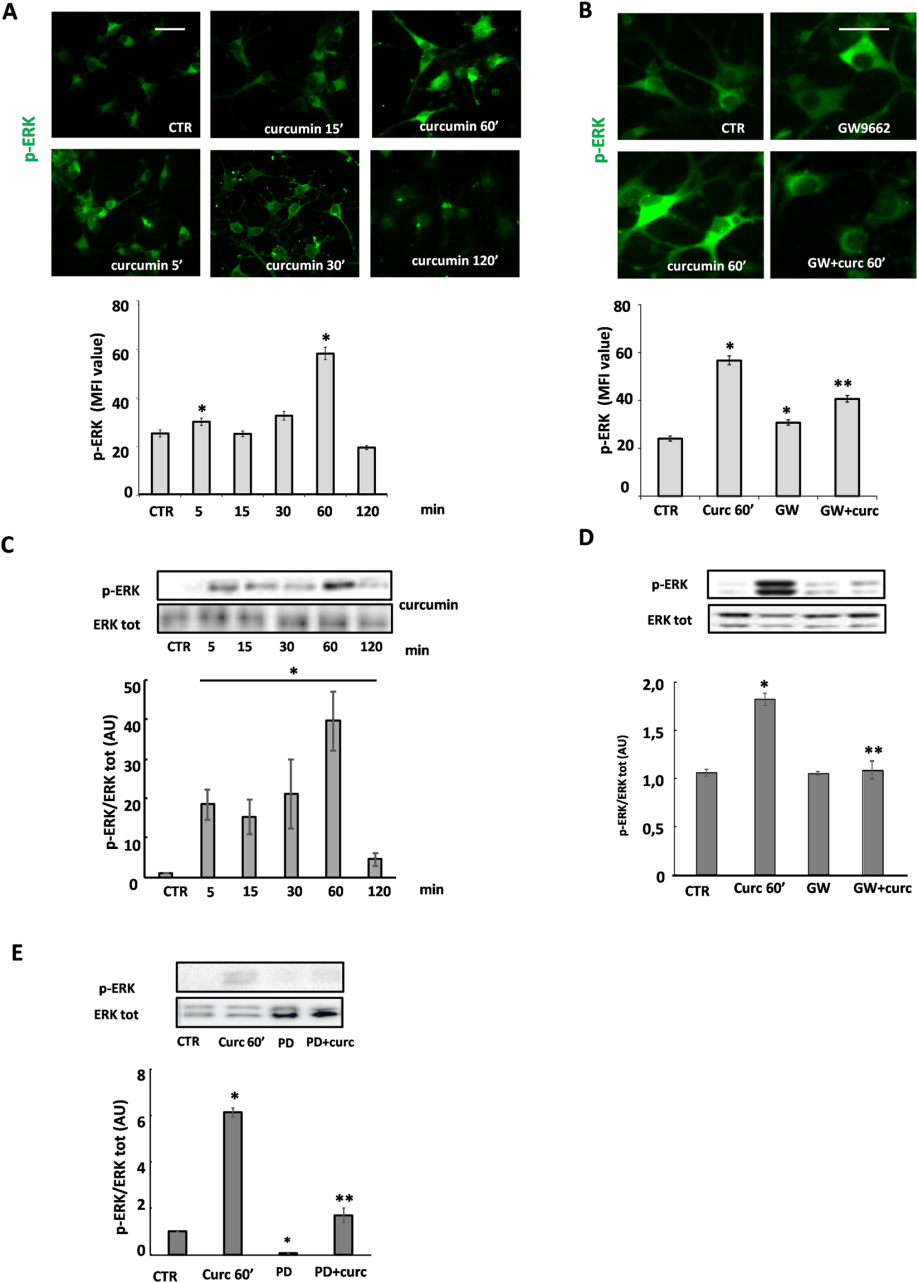
Curcumin modulates phosphorylation of ERK by involving PPAR-γ activation. Immunofluorescence and western blot analysis of the time-course of 1 µM curcumin-induced ERK 1/2 phosphorylation are shown in (**A**) and (**C**). Representative photomicrographs of p-ERK immunofluorescence are shown in panel A, at the indicated time points. The upper panel in C shows a representative western blot of p-ERK and total ERK. To confirm the ERK 1/2 activation by curcumin (curc), OPs were pretreated for 30 min with 10 μM PD98059 (PD; ERK1/2 inhibitor) and p-ERK was evaluated by western blot (**E**). To evaluate the involvement of PPAR-γ in curcumin-induced ERK1/2 activation, OPs were pretreated with 1 μM GW9662 (GW; PPAR-γ antagonist; **B** and **D**). p-ERK was evaluated by IF (MFI value is shown in the lower panel in **B**. Data are mean ± SEM of n. 300 cells for condition) and by western blot (panel **D**; Data are means ± SEM of three independent experiments). *p < 0.0001 versus CTR; **p < 0.05 vs curc. Scale bar 30 µm. Image Lab 4.0 software (Bio-Rad, http://www.bio-rad.com/en-us/sku/1709690-image-lab-software) and Leica Application Suite Software (260RI, https: //www.leica-microsystems.com/products/microscope-software/details/product/leica-las-x-ls/) were used respectively for Wb and IF image acquisitions.

In OP cultures exposed to 1 μM curcumin, ERK1/2 phosphorylation was induced in a time-dependent manner with maximum levels of p-ERK1/2 at 60 min, as determined either by immunofluorescence and subsequent quantification of mean fluorescence intensity (MFI) or by western blot (Fig. 2A, C). Interestingly, the PPAR-γ antagonist GW9662 significantly prevented the phosphorylation of ERK1/2 induced by curcumin (Fig. 2B), suggesting that the effect of curcumin on p-ERK1/2 is, at least in part, PPAR-γ-dependent. The results were confirmed by western blot experiments (Fig. 2D). As expected, phosphorylation of ERK1/2 induced by curcumin was abolished by 10 μM PD98059, a selective, cell-permeable inhibitor of the MEK/ERK pathway (Fig. 2E).

### Curcumin induces the expression of PGC1-α and COX1

One of the outcomes of PPAR-γ activation is the promotion of mitochondrial functions, as already shown in a variety of cells, including OPs. To confirm the capability of curcumin-induced PPAR-γ activation to impinge on mitochondrial functions, we studied the expression of two genes: (i) the peroxisome proliferator-activated co-activator 1-alpha (PGC1-α), known to promote the biogenesis of mitochondria and the defenses against oxidative stress, whose expression is induced by PPAR-γ; (ii) cytochrome C oxidase subunit 1 (COX1), known as a core protein of the cytochrome C oxidase (Complex IV) of the mitochondrial respiratory chain, whose expression is regulated by PGC1-α.

**Figure 3:**
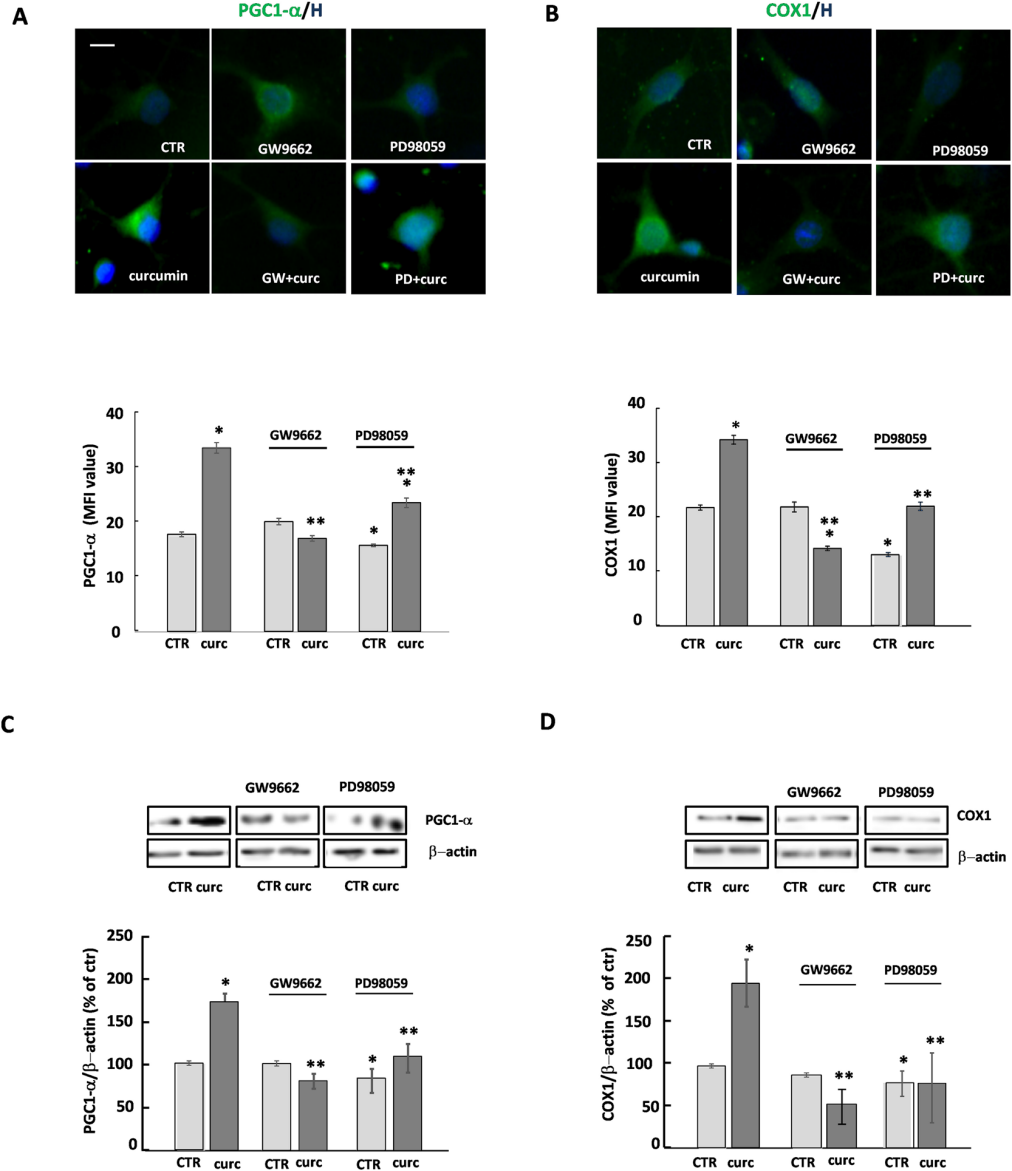
Curcumin induces the expression of PGC1-α and COX1. The expression of PGC1-α and COX1 was evaluated in OPs treated for 24 h with 1 μM curcumin (curc). To verify whether the effects of curcumin on PGC1-α and COX1 expression were PPAR-γ- and/or ERK-dependent, OPs were pretreated for 30 min with 1 μM GW9662 (GW; PPAR-γ antagonist) or 10 μM PD98059 (PD; ERK1/2 inhibitor). Representative photomicrographs of PCG1-α (panel **A**; green) or COX1 (panel **B**; green) are shown. Nuclei were stained using the Hoechst 33258 nuclear fluorochrome (blue, H). Mean fluorescence intensities (MFI value) are shown in the lower respective panels. Data are means ± SEM of 200–250 cells for condition. Western blot of PCG1-α and COX1 expression are shown in **C** and **D**, respectively. Representative western blot from same gel of PCG1-α or COX1 and β-actin (used as an internal control) are shown. Data are means ± SEM of three independent experiments (*p < 0.001 vs. CTR; **p < 0.01 vs .curc). Scale bar 10 µm. Image Lab 4.0 software (Bio-Rad, http://www.bio-rad.com/en-us/sku/1709690-image-lab-software) and Leica Application Suite Software (260RI, https: //www.leica-microsystems.com/products/microscope-software/details/product/leica-las-x-ls/) were used respectively for Wb and IF image acquisitions.

After 24 h of treatment, curcumin increased the levels of PGC1-α and COX1, an effect prevented by the presence of GW9662, confirming, also in this case, the effect of curcumin to be PPAR-γ-dependent (Fig. 3). Regarding ERK1/2 activation, the reduced expression of PGC1-α by PD98059 suggests that ERK1/2 is probably implicated in the modulation of the expression of PGC1-α. However, the strong reduction of COX1 expression of by PD98059 even in CTR condition does not allow conclusive speculation on the involvement of ERK1/2 in curcumin-dependent induction of COX1 (Fig. 3).

### Curcumin prevents TNF-α-induced harmful effects

**Figure 4:**
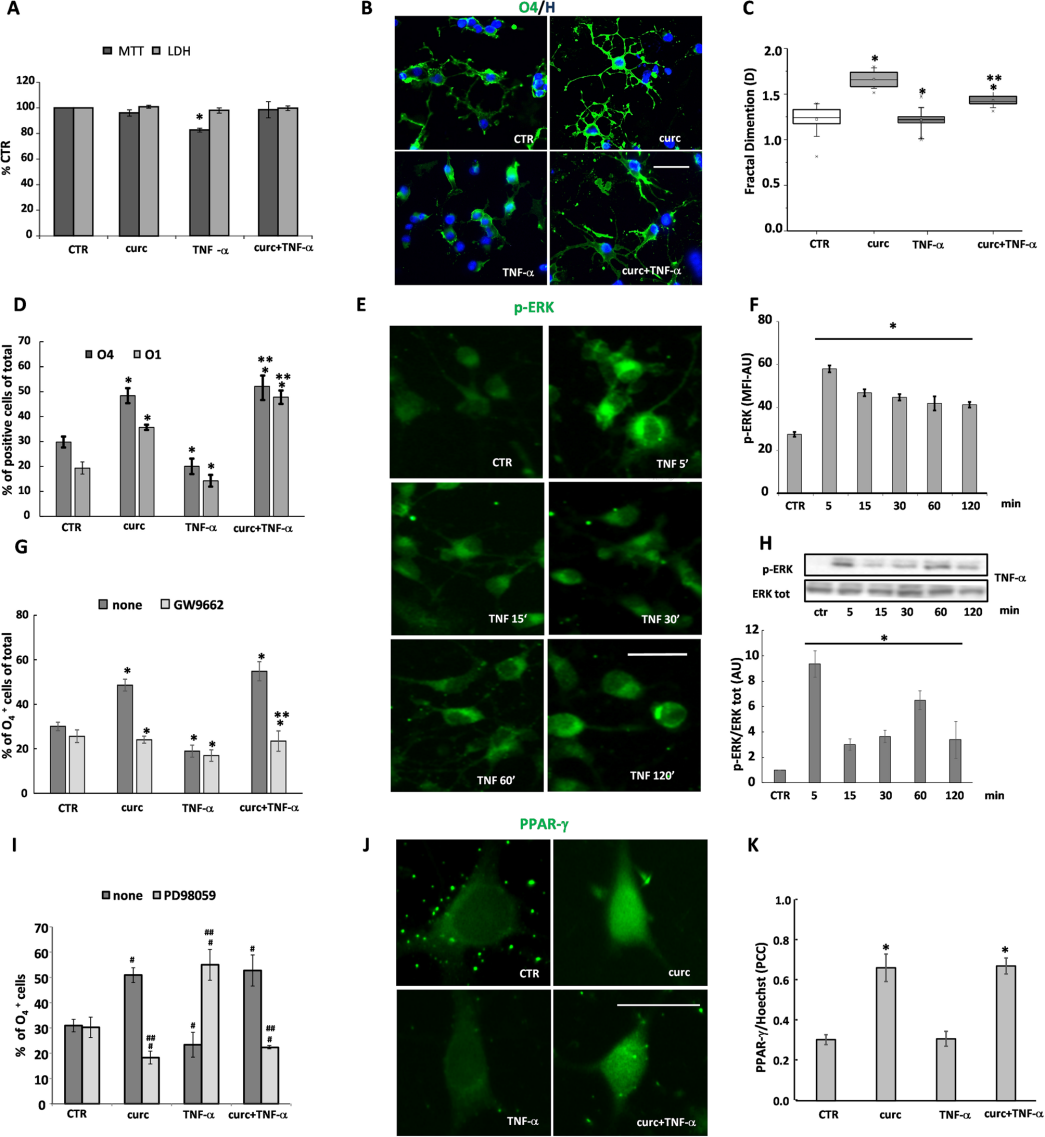
The detrimental effects of TNF-α on OP differentiation are counteracted by curcumin. Cells were treated with 10 ng/ml TNF-α for 24 h alone or in the presence of 1 μM curcumin (curc). OP viability was evaluated for MTT assay and LDH production. The values (means ± SEM) from 3–5 independent experiments run in triplicate are shown in (**A**). OPs were immunostained for O_4_ and O_1_, and nuclei were stained using the Hoechst 33258 nuclear fluorochrome (blue, H). Representative immunofluorescence images are shown for O_4_ (panel **B**, green). Differences on morphologies was evaluated by Fractal Dimension (D) value, the alteration of cellular complexity plotted in (**C**). Values represent at least n = 40 cells analyzed from three independent preparations. Cells were counted and percentages of O_4_- and O_1_-positive cells shown in (**D**). Data are mean ± SEM of 10 microscopic fields per coverslip prepared in duplicate for each condition from 3 to 5 independent experiments. *p < 0.005 versus CTR, **p < 0.0001 versus TNF-α. The percentages of O_4_- and O_1_-positive cells were also evaluated after pre-treatment with PPAR-γ antagonist GW9662 (GW) (G). To monitor nuclear translocation, OPs treated for 1 h with curcumin and/or with TNF-α were marked with anti-PPAR-γ antibody (green) (J). Co-localization with corresponding Pearson’s correlation coefficient (PCC), where PCC = 1 indicates complete co-localization and PCC = 0, absent co-localization, is shown in K. Panels E–H show immunofluorescence and western blot analysis of the time-course of TNF-α-induced ERK 1/2 phosphorylation. Data are mean ± SEM of 200–250 cells for condition. * *p* < 0.00001 versus CTR. OPs treated for 24 h with curcumin and/or TNF-α or in the presence of 10 μM PD98059 (PD) for 24 h were processed to verify the OP differentiation by evaluating the O_4_ expression (I). The percentage of O_4_ positive cells were calculated on the cell total number counted for each condition (means ± SEM, n = 3 independent experiments run in duplicate; ^#^*p* < 0.0001 vs. CTR, ^##^*p* < 0.05 vs own control). Scale bar 30 µm. Image Lab 4.0 software (Bio-Rad, http://www.bio-rad.com/en-us/sku/1709690-image-lab-software) and Leica Application Suite Software (260RI, https: //www.leica-microsystems.com/products/microscope-software/details/product/leica-las-x-ls/) were used respectively for Wb and IF image acquisitions.

It is known that TNF-α is capable of influencing proliferation, differentiation, and death in several cell types^23–25^, including OLs^18,26^. In this study, we wanted to determine the capability of curcumin to counteract the effect of TNF-α on OP differentiation and mitochondrial functions. We demonstrated that curcumin could prevent the adverse effects of TNF-α on viability (Fig. 4A) as well as on maturation of OPs (Fig. 4B, D), as indicated by the higher number of O_4_ and O_1_ positive cells as compared to TNF-α treated cultures. Curcumin also affected cell morphology in TNF-α treated OPs, and partially restored branch ramification (Fig. 4B, C). In the presence of GW9662, curcumin was no longer able to prevent the maturational arrest induced by TNF-α (Fig. 4G). Worth noting, curcumin induced the nuclear translocation of PPAR-γ also in the presence of TNF-α (Fig. 4J, K), further supporting the involvement of PPAR-γ activation in the protective effect of curcumin against TNF-α toxicity.

Besides, we demonstrated that TNF-α treatment induced a time-dependent increase of p-ERK, peaking at 5 min and then decreasing to a steady level that remained higher than in unstimulated cells up to 120 min, as revealed by MFI and by western blot analyses (Fig. 4E, F and H). Pretreatment with PD98059 was capable of counteracting OP differentiation induced by curcumin, either in the presence or absence of TNF-α, (Fig. 4I), suggesting that activation of ERK1/2 is involved in the processes triggered by both PPAR-γ and TNF-α.

**Figure 5:**
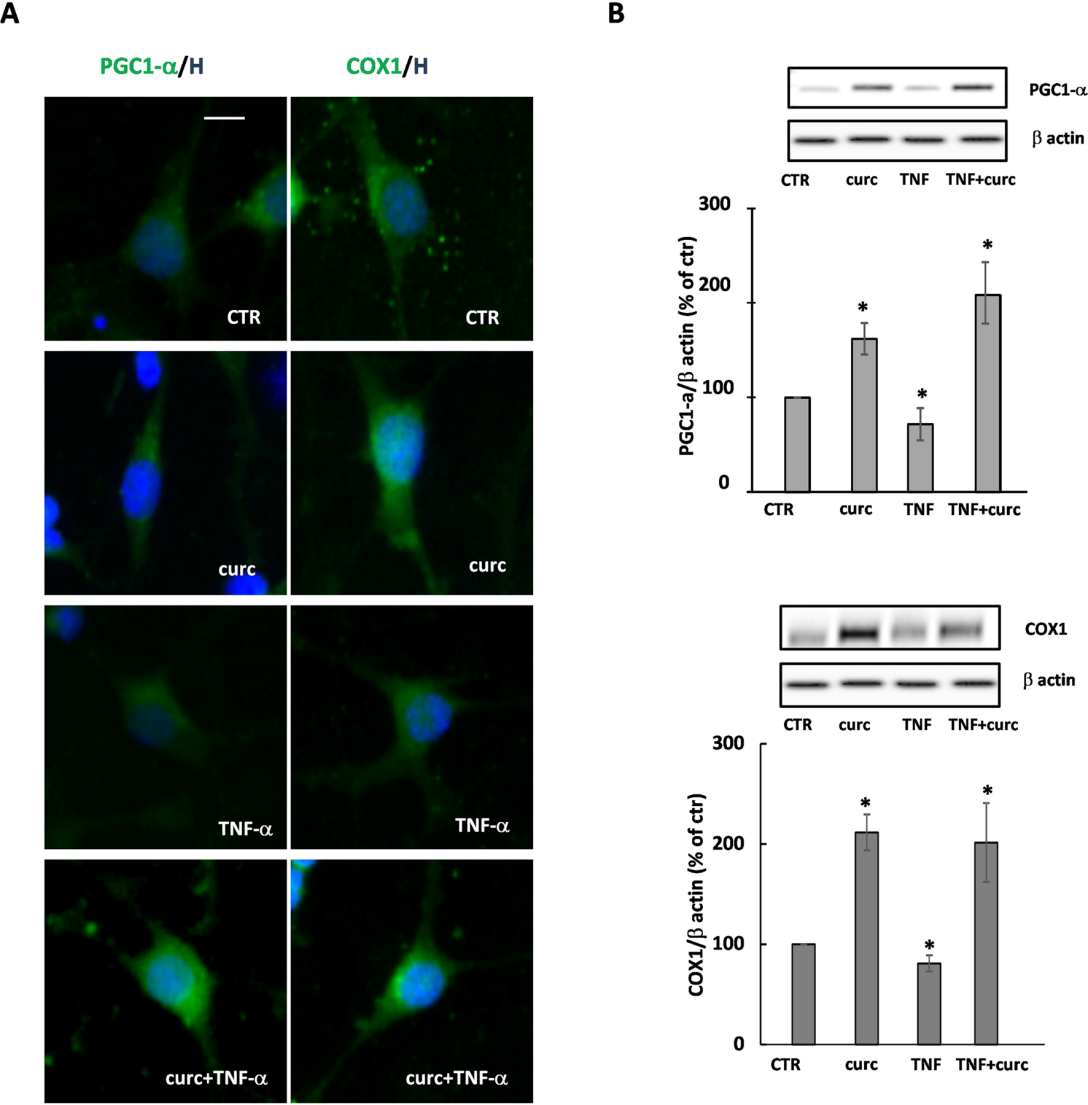
Curcumin protects from the effect of TNF-α on PGC1-α and COX1 expression. PGC1-α and COX1 expression levels were evaluated in cells treated for 24 h with curcumin (curc) and/or TNF-α. Representative photomicrographs are shown (**A**). Nuclei were stained using the Hoechst 33258 nuclear fluorochrome (blue, H). Representative western blot of PCG1-α or COX1 and β-actin (used as an internal control) are shown (**B**). The intensities of bands corresponding to PCG1-α or COX1 and β-actin were measured by densitometric analysis and the ratio PCG1-α/β-actin or COX1/β-actin is given as percentage on untreated cells. Data are means ± SEM of three independent experiments *p < 0.05 versus CTR, **p < 0.005 versus curc + TNF-α. Scale bar 10 µm. Image Lab 4.0 software (Bio-Rad, http://www.bio-rad.com/en-us/sku/1709690-image-lab-software) and Leica Application Suite Software (260RI, https: //www.leica-microsystems.com/products/microscope-software/details/product/leica-las-x-ls/) were used respectively for Wb and IF image acquisitions.

In agreement with the literature, we observed that TNF-α caused a mild, although significant, decrease of the levels of both PGC1-α and COX1 proteins, accentuating the toxic effect of TNF-α on mitochondria. When TNF-α was co-applied with curcumin, which alone increased COX1 and PGC1-α, the levels of the two proteins remained higher than in control as shown by immunofluorescence and western blot analyses (Fig. 5A, B, respectively). Together, the protective effect of curcumin on metabolic activity (MTT), PGC1-α and complex IV protein COX1 levels, support a protective effect of curcumin on mitochondrial functions.

## Discussion

In the present study, we tested if curcumin, acting as a PPAR-γ agonist, could favor the differentiation of OPs and their recovery from the damage caused by the pro-inflammatory cytokine TNF-α. Our final goal was to find experimental evidence to support the suggested therapeutic use of curcumin in myelin diseases, such as multiple sclerosis^27,28^.

We observed that curcumin favors the differentiation of OPs, as indicated by the increased expression of markers typically associated with different development stages. In particular, we assessed O_4_ and O_1_, two developmental markers whose presence is related to postmitotic commitment/initiation of terminal OL differentiation, and myelin basic protein (MBP), a late marker of OL maturation. Furthermore, cell morphology was more complex in curcumin-treated than in untreated cells, in keeping with cell maturation.

Curcumin-induced differentiation is consistent with previous studies showing curcumin action on neuronal differentiation and neurite outgrowth of PC-12 cells in combination with NGF^29^, and on the expression of differentiation genes in cells of thyroid carcinoma inducing G2/M arrest, apoptosis, and inhibition of NF-κB^30^.

Previous studies from our group have shown that PPAR-γ is constitutively expressed in OPs^14,20^ and that its activation by specific agonists promotes OP differentiation to mature OLs. Since curcumin is known to induce the activation of PPAR-γ in numerous cellular systems^31–33^, we hypothesized that similarly, the effects induced by curcumin on OPs could be mediated by PPAR-γ activation. In keeping with the hypothesis, we confirmed that curcumin activates PPAR-γ in OPs, by showing curcumin-dependent nuclear translocation of PPAR-γ, which was inhibited by the specific PPAR-γ antagonist GW9662.

The mechanism of action of PPAR-γ is mainly related to its ability to act as a ligand-dependent transcription factor. However, in recent years it has been highlighted how PPAR-γ can also act through "non-genomic" mechanisms. Once activated, PPAR-γ can physically interact with some proteins among which protein kinases, thereby modulating their activities. Among the protein kinases regulated by PPAR-γ is ERK1/2^21,34^.

ERK1/2 control the differentiation processes of different cell types. In the OL lineage activation of ERK1/2 is crucial for the transition from OPs to immature OLs^22,35^. Recently, we have shown that both synthetic (pioglitazone) and natural (the omega-3 PUFA DHA) PPAR-γ agonists induce ERK1/2 phosphorylation and that the activation of this molecular signaling contributes to OL differentiation and survival^17^. Here we show that curcumin induces the phosphorylation of ERK1/2, although with different kinetics as compared to those observed for pioglitazone and DHA^17^. ERK1/2 phosphorylation was partially inhibited by GW9662 pretreatment, demonstrating that the effect of curcumin is at least partly dependent on the activation of PPAR-γ. Indeed, a direct activation, i.e. phosphorylation, of ERK 1/2 by curcumin could also occur, as previously shown^36^.

Another goal of our study was to test whether curcumin could protect OP cultures from an inflammatory insult, as shown for other PPAR-γ agonists^16,17^. We mimicked the inflammatory condition by treating OPs with a subtoxic dosage of TNF-α (10 ng/ml)^18^, a pro-inflammatory cytokine involved in demyelinating diseases such as multiple sclerosis^26^. The addition of TNF-α in OP cultures inhibits their differentiation, probably through an adverse action on mitochondrial functions and Ca^2+^ signals^18,37^.

Among its numerous effects, TNF-α has been shown to regulate the activity of PPAR-γ negatively^38,39^. We observed that in the presence of TNF-α, curcumin induces the translocation of PPAR-γ and counteracts in a PPAR-γ-dependent manner, the maturational arrest of OPs triggered by the cytokine.

Both curcumin and TNF-α induced ERK1/2 phosphorylation, but with different kinetics, being faster for TNF-α than for curcumin (maximum level after 5 min and 60 min, respectively). The different kinetics of ERK1/2 phosphorylation^40^, could probably explain the opposite effects on OP differentiation by TNF-α and curcumin.

A large body of evidence supports the need for a proper mitochondrial function for myelination to occur. These organelles play fundamental roles in OL differentiation^16,18,37^. They are a source of energetic supply and a site where peculiar synthetic activities take place, such as part of the synthesis of cholesterol, one of the major constituents of myelin. We focused our attention on two proteins particularly significant in mitochondrial biogenesis: PGC1-α, which participates in the control of the transcription of genes involved in mitochondrial biogenesis, cholesterol synthesis and MBP gene expression^41^, and COX1, a core subunit of cytochrome C oxidase (Complexx IV) of the respiratory chain, whose expression is controlled by PPAR-γ through PGC1-α^42^. As for other PPAR-γ agonists or partial agonists, curcumin was able to increase their expression, by a PPAR-γ-dependent mechanism. Inhibition of ERK1/2 phosphorylation partially inhibited the effect of curcumin on PGC1-α, but not that of COX1. As expected, TNF-α caused a moderate decrease of the expression levels of both PGC1-α^10,18,24,43^ and COX1^18,25,44^, which was counteracted by curcumin. A protective effect of curcumin against mitochondrial injury and OL/neuronal apoptosis has been shown in experimental autoimmune encephalomyelitis (EAE), an animal model of MS^6^, suggesting that a similar mechanism could occur also in TNF-α treated OPs.

**Figure 6:**
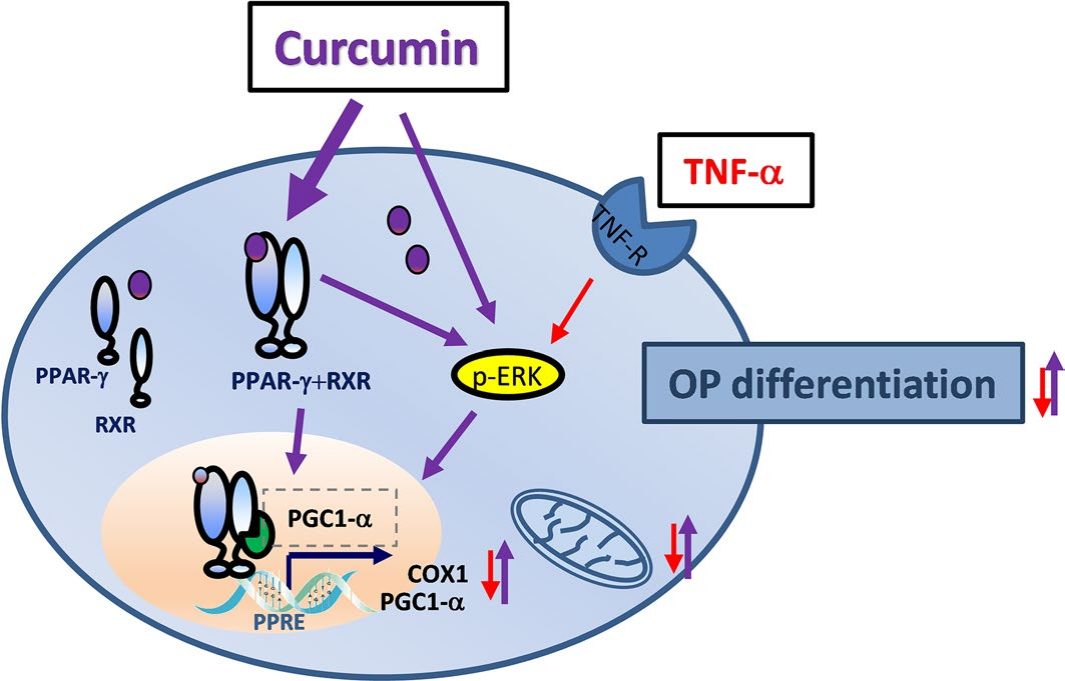
Graphical representation of curcumin effects on OPs.

In summary, our study highlights the capability of curcumin of promoting the differentiation of OPs, even in an inflammatory context, through multiple mechanisms involving PPAR-γ and ERK1/2 activation, possibly through mechanisms affecting mitochondria (Fig. 6).

Despite the wide range of beneficial effects of curcumin, its therapeutic use is limited by its poor water solubility, poor absorption from gastrointestinal mucosa, low stability in blood and rapid hepatic metabolism^45^. Several approaches have been taken to enhance its bioavailability, including the use of adjuvants (such as piperine‚ quercetin or silibinin), of complexes of curcumin with phospholipids, polysaccharides or proteins, and, more recently encapsulation in nanoparticles^46^.

Promising in vivo studies and clinical trials support the use of curcumin nanoparticle (nanocurcumin) in CNS diseases. Polymerized nanocurcumin was effective in controlling inflammation in EAE. Moreover, curcumin-loaded nanoparticles promoted myelin repair in a model of demyelination induced by lysolecithin in the rat corpus callosum^47^. Of note, nanocurcumin was recently shown to reduce neuroinflammation and restore dysregulated Treg functions in a clinical trial enrolling 50 relapsing–remitting MS subjects, after six months of treatment^48,49^.

The picture emerging is of growing interest for curcumin, with the possibility of expanding the use of an old natural compound through new and more efficient formulations in different pathological situations^50^.

The present study, based on a well-established in vitro model, provides evidence for a further mechanism of action of curcumin, by showing its ability to activate PPAR-γ in OPs and promote their differentiation to OLs and, in turn, myelin formation. Further studies on more complex models of myelination will deepen our knowledge of curcumin and provide mechanistic evidence besides its well-known anti-inflammatory properties in support of the proposed utilization of curcumin in demyelinating pathologies^23,27^.

## Methods

All the experiments have been carried out on purified cell cultures. The procedure for obtaining purified cell cultures has been approved by Ministry of Health (authorization number: 152/2016-PR) based on the European Communities Council Directive N. 2010/63/EU and the Italian Law Decree n° 26/2014. Moreover, all experiments were conducted following the relevant guidelines and regulations.

### Cell cultures

Purified cultures of OPs were obtained as previously described^14^. Forebrains from postnatal day 1 Wistar rats (RRID: RGD_13508588; from Charles River, Lecco, Italy) were mechanically dissociated, and the cells obtained were grown for 10 days in Dulbecco's modified Eagle's medium (DMEM with high glucose), supplemented with 10% foetal bovine serum (Gibco, ThermoFisher Scientific, Inc.) on poly-L-lysine coated 60-mm-diameter culture dishes. OPs growing on top of mixed glial monolayers were mechanically detached from the mixed cultures. A mechanical detachment was followed by 1 h incubation at 37 °C in culture flasks to remove adhering cells and thus minimize contamination by microglia. Then cells were seeded at the density of 6 × 10^4^ cells/cm^2^ into poly-L-lysine-coated 35-mm diameter plastic culture dishes or 96 well plates. After 2 h, the culture medium was replaced with a chemically defined serum-free medium consisting of DMEM/HamF12 (4:1) supplemented with 5.6 mg/ml glucose, 5 µg/mL insulin, 100 µg/mL human transferrin, 100 µg/mL bovine serum albumin, 0.06 ng/mL progesterone, 40 ng/mL sodium selenite, 16 µg/mL putrescine, 50 U/mL penicillin, 50 µg/mL streptomycin, 2 mmol/L glutamine,10 ng/ml human recombinant PDGF-AA and 10 ng/ml human recombinant bFGF (PeproTech EC, Ltd, UK). Immunocytochemical analysis with LB_1_ and O_4_ antibodies revealed a pure population (around 98%) of cells of the OL lineage. Cell culture reagents were from BioWest (Fla, USA) or Invitrogen (Milan, Italy); other chemicals were from Sigma-Aldrich Italia (Milan, Italy). The cells were treated at 1DIV after seeding, and the experiments were done at 2DIV (if not otherwise stated in the figure legend).

### Experimental design

Purified oligodendrocyte precursors (OPs) were maintained in a chemically defined serum-free medium for all experiments. At 1 DIV, 24 h after plating, OPs were stimulated by adding different drugs, without changing the medium. Curcumin (1 or 5 μM) was added alone or in the presence of TNF-α (10 ng/ml). Depending on the parameter to be studied, the experiments were carried out for short time (from 5 to 120 min), or for longer times for 24 h (2 DIV) or 7 DIV (see details in the results section and the legends). When GW9662 (1 μM) or PD98059 (10 μM) were used, the OPs were pretreated for 30 min before the addition of other drugs. After the experimental time, cells were treated according to the different procedure, as described later. Conditioned media were collected, centrifuged, filtered, and stored at − 80 °C until tested.

### Cell viability

The ability of cells to reduce 3-(4,5-dimethyl thiazol-2-y1)-2,5-diphenyl tetrazolium bromide (MTT, Sigma, Munich, Germany) was assessed as an index of cell metabolic activity, as previously described^14^. Briefly, MTT was added at a final concentration of 0.25 mg/ml during the last 4 h of incubation. The medium was then removed, and 100 μl DMSO added to each well to dissolve the dark blue crystals. The plates were then read on a microplate reader, using a test wavelength of 570 nm and a reference wavelength of 630 nm. The release of the cytosolic enzyme lactate dehydrogenase (LDH) was used to evaluate the integrity of the membrane, by verifying its presence in the culture supernatant using a colorimetric cytotoxicity assay kit (Roche Diagnostics MI, Italy). Apoptotic cells were evaluated observing the changes of nuclei morphology. Cells were exposed to 1 µg/mL of Hoechst 33258 for 60 min to 37 °C, rinsed with phosphate‐buffered saline (PBS), fixed with 4% paraformaldehyde. Nuclear morphology was visualized using a fluorescence microscope. To detect and quantify, at the single-cell level, DNA strand breaks generated during apoptosis we used terminal deoxynucleotidyl transferase (TdT)-mediated dUTP-fluorescein nick end-labeling (TUNEL) staining. An in-situ cell death detection kit was used (Boehringer Mannheim, Indianapolis, IN) and TUNEL reaction was analyzed by fluorescence light microscopy.

### Immunofluorescence

Cell differentiation was evaluated by immunofluorescence for membrane surface antigens, using the monoclonal immunoglobulin M, O_4_ and O_1_, as previously described^17^ (IgM, hybridoma supernatants, custom made, or Millipore, Italia; 1:5 or 1:100, (Cat#MAB344 clone 59 Lot#2343576), respectively). For immunofluorescence of intracellular antigens, cells were fixed in 4% paraformaldehyde for 10 min at room temperature (RT). After fixation and permeabilization with 0.2% Triton X-100 for 10 min at RT, the cells were pre-incubated with 3% BSA in 0.1% Triton X-100/PBS solution for 1 h at RT and then incubated overnight at 4 °C with rabbit polyclonal anti-COX1 (i.e. subunit 1 of cytochrome C oxidase, 1:100, Cat# ab45918, RRID:AB_944283, Abcam, UK), anti-PGC1-α (1:50, H-300, sc-13067, RRID:AB_2166218, Santa Cruz Biotechnology, Inc, USA), anti-PPAR-γ (1:100, Cat# sc-7196, RRID:AB_654710, Santa Cruz Biotechnology, Inc, USA) or monoclonal anti-MBP (1:100, Cat#MAB382, RRID: AB_94971, Millipore, Milano, Italia) in the same pre-incubation solution. As secondary antibody, fluorescein-conjugated goat anti-mouse IgM/IgG or anti-rabbit IgG (1:200, Jackson ImmunoResearch Laboratories, Inc, West Grove, PA) were used. For p-ERK labelling the cells were incubated with 2.5% horse serum and then with an antibody against p-ERK (1:100, #4370, lot #12, Cell Signaling Technologies) for 1 h at 37 °C; after washing they were incubated first with biotinylated goat α-rabbit (1:200, Vector Laboratories, Inc, Burlingame, CA) for 45 min at 37 °C and then with Alexa 488 (1:200, Jackson ImmunoResearch Laboratories, Inc, West Grove, PA) for 1 h at RT. Validation data for the antibodies are available from the companies. Nuclei were stained using Hoechst 33258 (5 µg/ml for 20 min, Sigma, Munich, Germany). Coverslips were mounted with Vectashield Mounting Medium (Vector Laboratories, Burlingame, CA) and examined using a Leica DM4000B fluorescence microscope equipped with a DFC420C digital camera and Leica Application Suite Software (260RI, RRID:SCR-013673, URL: https://www.leica-microsystems.com/products/microscope-software/details/product/leica-las-x-ls/) for image acquisition (Leica, Wetzlar, Germany). The experiments were repeated on a minimum of three independent cultures of OPs. For cell imaging, at least six-eight different fields in each coverslip were captured, and all cells in the pictures were analyzed for their fluorescence content. Image acquisition was maintained in the same setting under various experimental conditions. Exposure parameters, saturation, time, and gain were set at the beginning of every single group of experiments. For the images obtained from fixed cells, the acquisition information was space resolution 2592 × 1944, pixel dimensions 0.340 µm/pixel, and image bit depth 8 bit × 3 channels. The fluorochromes used are in the range of wavelengths of 495–520 for L5 (green), 552–663 for Y3 (red) and 430–474 for A4 (blue). Every image was acquired with a maximum resolution of 400 DPI. The scale bars are indicated in each figure legend. All image analyses were conducted using NIH ImageJ software (http://rsb.info.nih.gov/ij/). Mean threshold fluorescence intensity (MFI) within a region of interest, delineated by single-cell profile, was used to compare expression levels of specific markers. Always using NIH ImageJ, co-localization of specific markers was analyzed by Pearson's correlation coefficient^51^. Fractal dimension (D) analysis was applied to evaluate the morphological complexity of cells. A numerical value close to 1 for cells with low morphological complexity (essentially bipolar cells) and near 2 for those with high complexity (highly branched cells or with a bi-dimensional planar structure^52^, was applied. This procedure was performed for at least 40 cells per condition for each independent experiment (n = 3 independent experiments). Alternatively, the cells were counted in 10 microscopic fields of 0.18-mm^2^ per coverslip prepared in duplicate for each condition from at least 3 independent experiments.

### Western blot analysis

Cells were homogenized on ice in RIPA buffer (phosphate-buffered saline, 1% NP-40, 0.5% sodium deoxycholate, 0.1% sodium dodecyl sulphate, protease inhibitors) and centrifuged at 12000 g for 20 min, 4 °C. Equal amounts of protein (40 μg for COX1, MBP and PCG1-α, and 10 μg for p-ERK/total ERK) were separated by electrophoresis on 8–12% SDS-PAGE gels and transferred onto polyvinylidene difluoride membranes by electroblotting for 1 h at 4 °C. The membranes incubated for 1 h in T-TBS (50 mM Tris–HCl, 150 mM NaCl, 0.05% Tween-20, pH 7.4), with 5% BSA in T-TBS. Blots were incubated overnight at 4° C with the following antibodies: rabbit anti-COX1 or anti-PCG1-α (1:400 Abcam, Cambridge UK), monoclonal anti-MBP (1:600) or rabbit anti-p-ERK (1:2000 Cell Signaling Technologies), and rabbit anti-ERK (1:1000, Cell Signaling Technologies) or mouse anti-β-actin (1:20,000, Cat#A2228, RRID: AB_476697 Sigma-Aldrich), which were used as an internal control. After incubation with HRP-conjugated antibodies (Jackson Immunoresearch), immunoreactive bands were revealed directly by ChemiDoc XRS imaging system (Bio-Rad, Italy) and were analyzed using Image Lab 4.0 software (Bio-Rad, RRID: SCR-014210, URL: http://www.bio-rad.com/en-us/sku/1709690-image-lab-software).

### Statistical analysis

Data were expressed as means ± SEM. Statistical significance was evaluated using Student's t-test or one-way ANOVA (Microsoft Windows Excel Office 365 and confirmed in Origin 6.0, OriginLab, Northampton, MA). Numbers of independent experiments are indicated in the figure legends; *p* < 0.05 was accepted as statistically significant. The experimental procedures and statistical analysis of the data presented in this study followed the methodologies and standards generally used in in vitro studies. In all experiments, the sources of variability, as well as any residuals deriving from random errors of the replicates, were well controlled, as demonstrated by the low SEM. Bonferroni correction was adopted to account for multiple comparisons. Normality of distribution was assessed by the Shapiro Wilk test (Microsoft Windows Excel Office 365 and confirmed by online software http://www.statskingdom.com)^53^.

## Supplementary Information

Supplementary Figures.
