# Supplementary material for: Curcumin promotes oligodendrocyte differentiation and their protection against TNF-α through the activation of the nuclear receptor PPAR-γ
Source: Sci Rep. 2021 Mar 2;11:4952. doi: 10.1038/s41598-021-83938-y (PMC7925682; doi:10.1038/s41598-021-83938-y)

Curcumin promotes oligodendrocyte differentiation and their protection against TNF-α through the activation of the nuclear receptor PPAR-γ

Antonietta Bernardo, Cristina Plumitallo, Chiara De Nuccio, Sergio Visentin & Luisa Minghetti

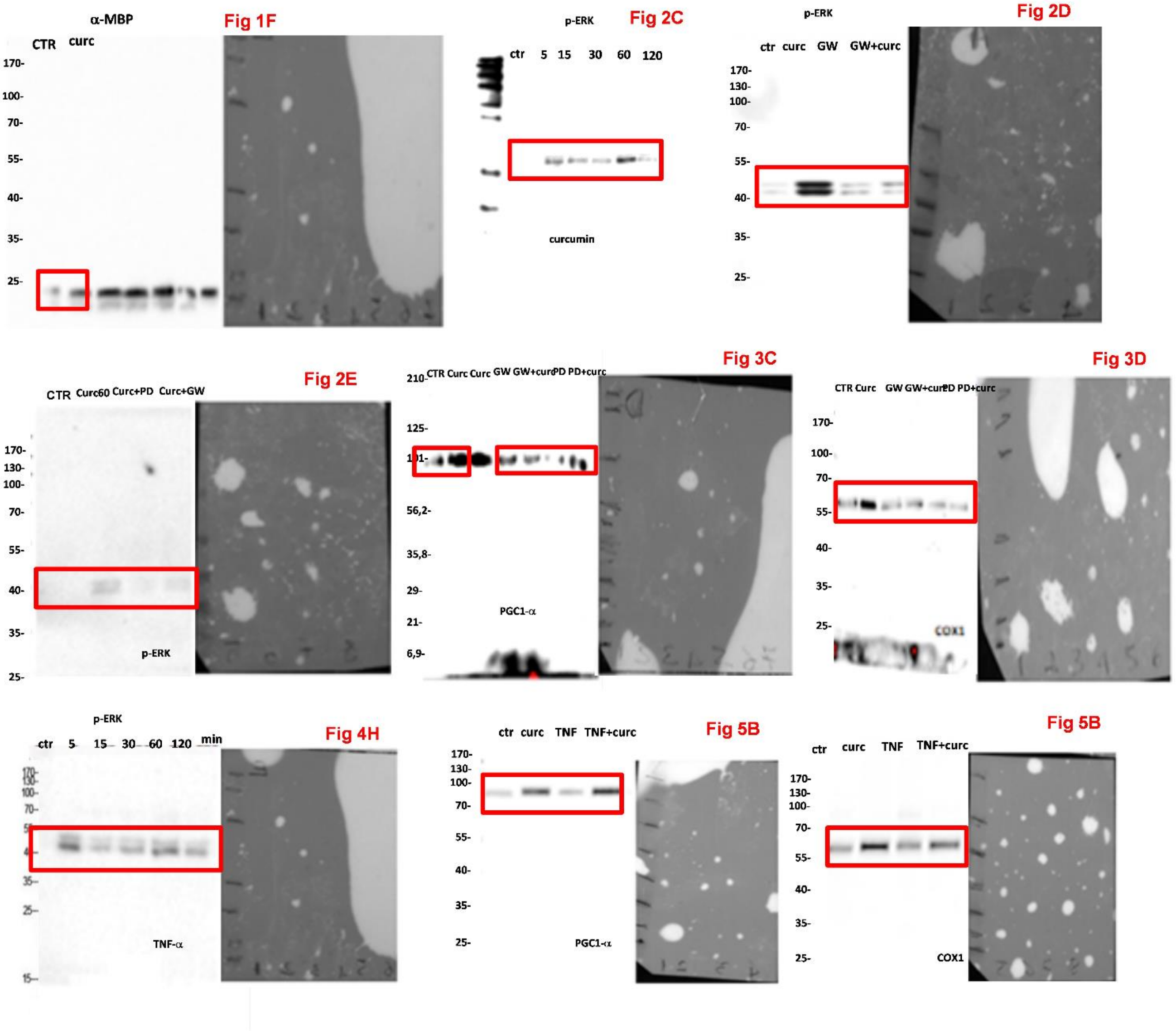

The images represent the full-length of western blots and respective membranes for MBP, p-ERK, PGC1-a and COX1 shown in the figures 1-5, for details see Figure legends.

Exposure images

Fig 1F MBP

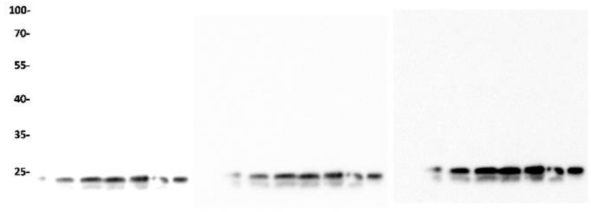

Fig 2C p-ERK

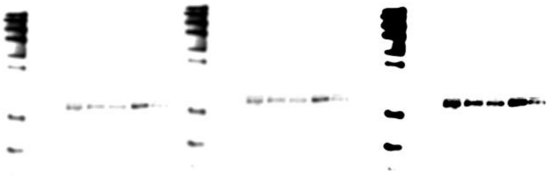

Fig 2D p-ERK

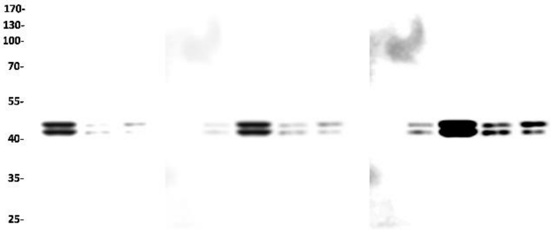

Fig 2E p-ERK

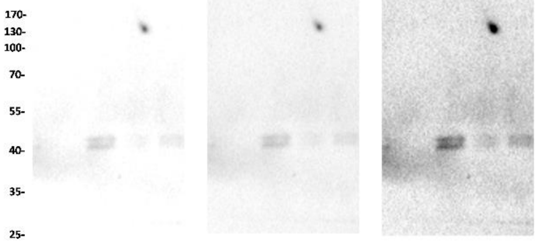

Fig 3C PGC1α

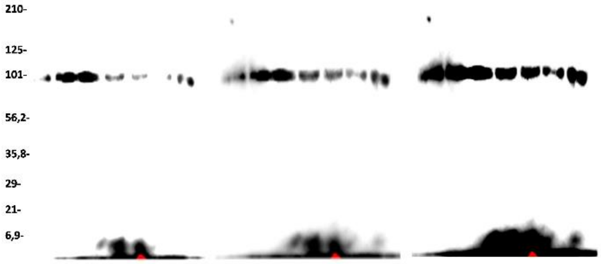

Fig 3D COX1

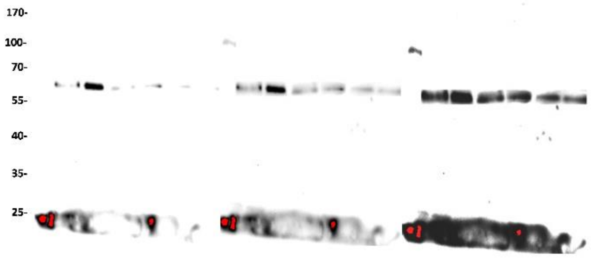

Fig 4H p-ERK

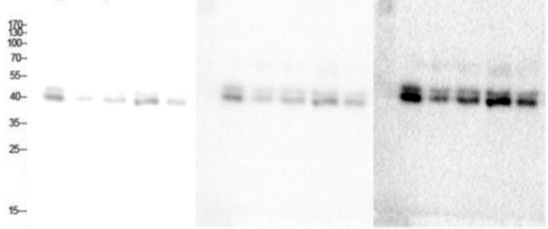

Fig 5B PGC1α

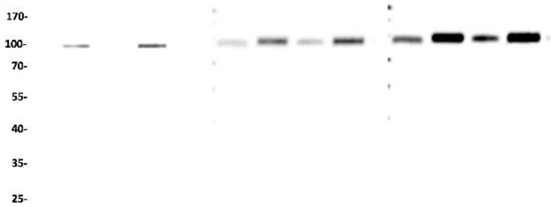

Fig 5B COX1

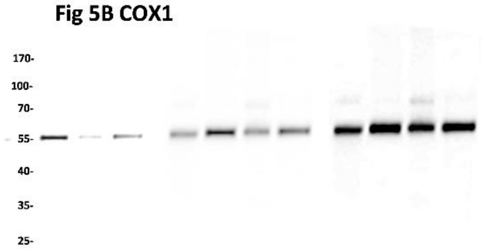

Fig 1F β-actin

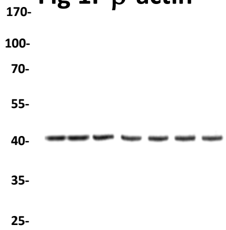

Fig 2C ERK tot

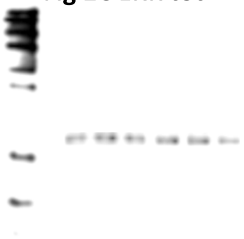

Fig 2D ERK tot

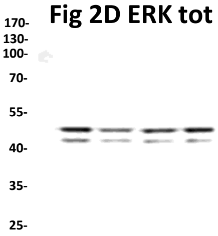

Fig 2E ERK tot

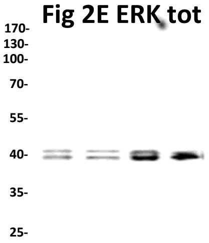

Fig 3C β-actin

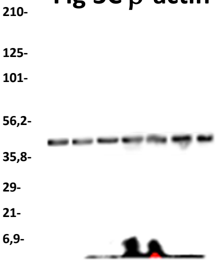

Fig 3D β-actin

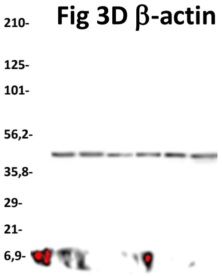

Fig 4H ERK tot

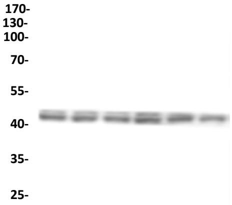

Fig 5B β-actin (PGC)

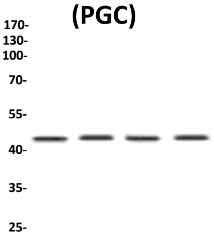

Fig 5B β-actin (COX)

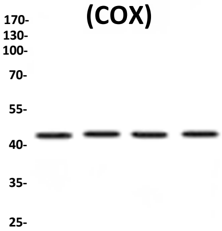

Different exposures

Fig 1F β-actin

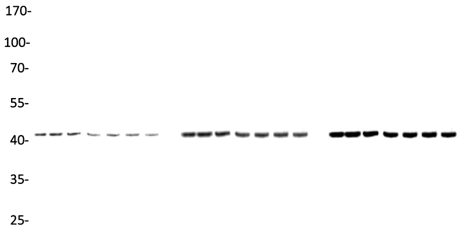

Fig 2C ERK tot

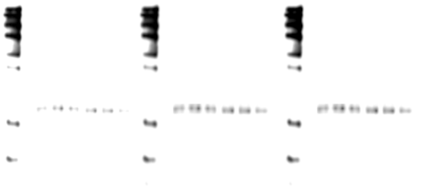

Fig 2D ERK tot

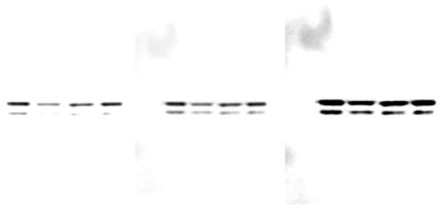

Fig 2E ERK tot

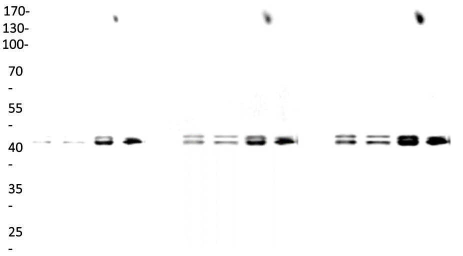

Fig 3C β-actin

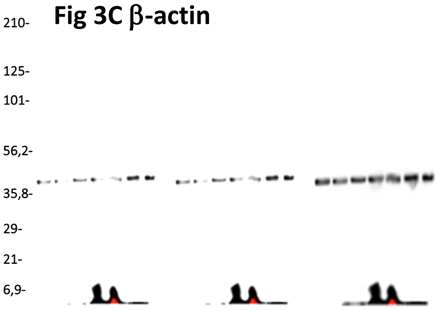

Fig 5B β-actin (PGC)

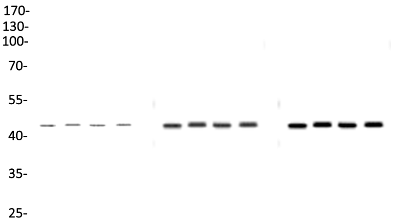

Fig 3D β-actin

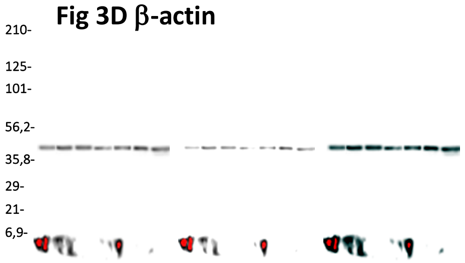

Fig 4H ERK tot

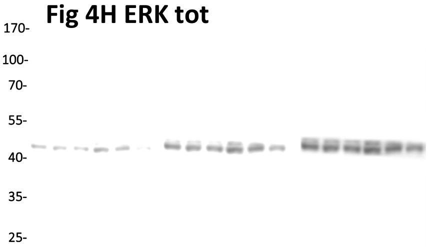

Fig 5B β-actin (COX)

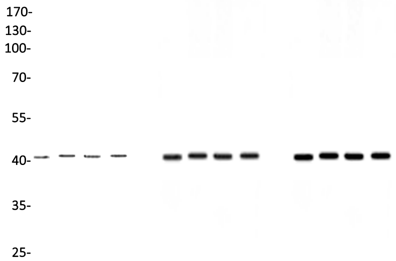

Supplement: Supplementary file 1 — Supplementary Figures. [file 41598_2021_83938_MOESM1_ESM.pdf]
